# How do socioeconomic attainment gaps in early mathematical ability arise?

**DOI:** 10.1111/cdev.13947

**Published:** 2023-05-30

**Authors:** Ella James‐Brabham, Toni Loveridge, Francesco Sella, Paul Wakeling, Daniel J. Carroll, Emma Blakey

**Affiliations:** ^1^ Department of Psychology University of Sheffield Sheffield UK; ^2^ Centre for Mathematical Cognition and Centre for Early Mathematics Learning, Department of Mathematics Education Loughborough University Loughborough UK; ^3^ Department of Education University of York York UK

## Abstract

Socioeconomic attainment gaps in mathematical ability are evident before children begin school, and widen over time. Little is known about why early attainment gaps emerge. Two cross‐sectional correlational studies were conducted in 2018–2019 with socioeconomically diverse preschoolers, to explore four factors that might explain why attainment gaps arise: working memory, inhibitory control, verbal ability, and frequency of home mathematical activities (*N =* 304, 54% female; 84% White, 10% Asian, 1% black African, 1% Kurdish, 4% mixed ethnicity). Inhibitory control and verbal ability emerged as indirect factors in the relation between socioeconomic status and mathematical ability, but neither working memory nor home activities did. We discuss the implications this has for future research to understand, and work towards narrowing attainment gaps.

**Abbreviations**
BPVS: British Picture Vocabulary Scale
CI: confidence interval
IMD: Index of Multiple Deprivation
SES: socioeconomic status

## INTRODUCTION

The development of early mathematical skills is of great importance—not simply for building more advanced mathematical skills (Watts et al., 2014), but also because it is a strong predictor of overall academic attainment (Duncan et al., 2007). Poor mathematical skills have consequences far beyond academic attainment, including negative associations with health, income, and quality of life (National Numeracy, 2015). There are large individual differences in mathematical ability, and one factor which predicts these differences is socioeconomic status (SES), for example, maternal education at age four predicts mathematical achievement at age 15 (Ahmed et al., 2018). SES refers to an individual's combined social and economic resources, and position within society (Duncan & Magnuson, 2012). By the time children begin formal education, SES disparities in mathematics are already apparent, with children from lower‐SES households having poorer mathematical ability, on average, than children from higher‐SES households (Sirin, 2005). These early SES disparities not only persist, but increase, over the duration of a child's schooling (Caro et al., 2009). This has profound consequences: in the United Kingdom, one in four adults is estimated to have a lower mathematical ability than is needed for everyday life (Organization for Economic Co‐operation and Development, 2013). It is therefore crucial to understand how SES disparities in mathematical ability first arise. Little is known about the mechanisms by which attainment gaps in mathematical ability emerge. This lack of understanding is a major obstacle to any attempt to narrow these gaps before they embed. Therefore, the present research aims to investigate multiple factors that might explain how SES attainment gaps in early mathematical ability arise.

When seeking to understand how SES influences mathematical ability, it is particularly important to focus on *early* mechanisms. This is because mathematical learning proceeds incrementally: each numerical principle helps to form the foundations for later, more advanced principles. If a child lacks foundational mathematical skills (e.g., one‐to‐one correspondence), they will have difficulty building more advanced mathematical knowledge (e.g., basic addition; Baroody et al., 2012). As a child falls behind in their mathematical learning, it becomes incrementally harder for them to catch up, resulting in an ever‐widening gap between the lowest‐ and highest‐ability children (Educational Endowment Foundation, 2017). Identifying the factors that give rise to this attainment gap will be essential for developing theoretical models that can be tested in longitudinal and intervention work, with the long‐term aim of narrowing the SES attainment gap.

There are many factors that might explain why SES gaps in early mathematics arise. These include variables that influence the context into which a child is born (e.g., poverty, parent health); variables relating to the home and school environment; and child‐level competencies (Ribner et al., 2019). Variables that influence the context in which a child is born are largely unchangeable without major, long‐term shifts in policy at a global or national level. In the present research, we focus on four home‐ or child‐level factors that may explain mathematical attainment gaps: working memory, inhibitory control, verbal ability, and home mathematical activities. There are three reasons in particular to focus on these factors. Firstly, all four factors have been found to relate to preschoolers' mathematical skills and each tends to show socioeconomic gradients. Secondly, these four factors reflect more proximal mechanisms that may directly explain SES attainment gaps. Thirdly, these home‐ and child‐level factors are often more malleable, and so provide plausible targets for any future interventions.

Working memory is the first proposed factor by which SES disparities in early mathematics may develop. Working memory is a core cognitive ability that enables us to maintain and manipulate information (Diamond, 2013). Working memory may support early mathematics by enabling children to retrieve numerical facts, and to maintain and process numerical information to successfully carry out mathematical operations. Working memory has been found to positively relate to preschoolers' mathematical ability (Blakey & Carroll, 2015; Blakey et al., 2020). In addition, environmental factors linked to SES—including stress, nutrition, and cognitive stimulation—are also linked to the development of brain areas responsible for higher‐order cognitive control, including working memory (Hackman & Farah, 2009). Indeed, working memory itself has been found to vary by SES, with children from higher‐SES families having better working memory, on average, than children from lower‐SES families (Lawson et al., 2018). Furthermore, recent work has identified that preschool working memory mediates SES attainment gaps in middle childhood (Waters et al., 2021). Therefore, working memory is an important variable to examine in its role in early socioeconomic attainment gaps in mathematics.

Inhibitory control is the second proposed factor by which SES disparities in early mathematics may develop. Inhibitory control enables us to suppress distractions and resist prepotent but incorrect responses (Diamond, 2013). Inhibitory control may support early mathematical skills by helping children to ignore distracting information while focusing on a mathematical problem, and helping them to suppress prepotent but incorrect strategies when solving a problem. A meta‐analysis found a medium effect size for the relation between preschoolers' inhibitory control and mathematical ability (Allan et al., 2014). Inhibitory control is a higher‐order cognitive process, and as such is influenced by environmental factors, which have socioeconomic gradients (Hackman & Farah, 2009). Indeed, inhibitory control varies by SES, with children from higher‐SES families having, on average, better inhibitory control than children from lower‐SES families (Blakey et al., 2020; Lawson et al., 2018). It is therefore plausible that inhibitory control plays a role in socioeconomic differences in early mathematical skills.

There has been some debate regarding whether working memory and inhibitory control should be considered separate facets of cognition (e.g., Wiebe et al., 2011), or be considered part of a broader single factor in early childhood (e.g., Miller et al., 2012). Pertinent to individual difference research, researchers have stressed the usefulness in looking at working memory and inhibitory control separately, given they often differentially predict academic outcomes (Lerner & Lonigan, 2014). As we were focused on understanding specific factors that may underpin attainment gaps, we rely more on the latter approach to enable us to say with greater specificity what factors may explain mathematical attainment gaps.

Verbal ability is the third proposed factor by which SES differences in early mathematical ability may develop. Socioeconomic disparities in verbal ability are well documented: from as young as 18 months of age, children from lower‐SES households have significantly fewer words in their vocabulary than children from higher‐SES households (Fernald et al., 2013). SES differences in verbal ability have been linked to both the quantity of words children hear in the home, and the quality of language interactions (Hoff, 2003). There has been a wealth of research demonstrating that children with higher verbal ability have more advanced reading skills (e.g., Duff et al., 2015) though less research has examined its role in mathematical skills. However, there is emerging evidence indicating that verbal ability may be important for early mathematical development. Conceptually, the development of mathematics is closely related to the development of verbal ability. A child's ability to map vocabulary to number marks the transition from non‐symbolic to symbolic number understanding (Xenidou‐Dervou et al., 2015). An example of this at the most basic level is that when children first learn the count sequence, they are essentially learning words with an arbitrary meaning. However, learning these words is essential for children to learn the cardinal principle (mapping number to quantity) which gives number words meaning and is a cornerstone of subsequent mathematical development (Wynn, 1995). Following this, children will begin to learn more specialist mathematical language, including comparative terms (e.g., “more” vs. “less”; “bigger” vs. “smaller”) and mathematical operators (e.g., “add”, “subtract”). This shows how verbal ability is directly intertwined with early mathematical learning. Indeed, LeFevre et al. (2010) identified verbal ability as a key pathway to mathematical development in children aged 4–6 years (see also Purpura et al., 2011). More recently, verbal ability has been found to mediate the relation between SES and mathematical ability (Slusser et al., 2019; von Stumm et al., 2020). While there is emerging evidence that verbal ability supports early mathematical ability, many studies tend to use receptive vocabulary as a marker of general cognitive ability. This makes it difficult to disentangle the roles of verbal ability and general cognitive ability. In order to understand whether verbal ability can explain mathematical ability above and beyond general cognitive ability, it is vital that studies control for general cognitive ability using an alternate variable to vocabulary, such as processing speed (Finkel et al., 2005). We opted to use processing speed in our study as it was age appropriate for young preschools (in contrast to IQ measures, which tend to be used from age five), and the task had minimal overlap with our key variables of interest.

Home mathematical activities are the fourth factor by which socioeconomic attainment gaps in early mathematical ability may develop. In recent years, there has been an increased focus on the role of the home environment in the development of mathematical skills, focusing on the *frequency* of the mathematical activities that parents do with their children in the home (Elliott & Bachman, 2018). When we look at frequency, an intriguing but inconsistent picture emerges: substantial variation is found in the frequency with which parents report that they engage in home mathematical activities (from every day to not at all), and this frequency sometimes relates positively to children's mathematical ability (e.g., Kleemans et al., 2012; Lefevre et al., 2009); sometimes negatively (Blevins‐Knabe et al., 2000; Ciping et al., 2015); and sometimes not at all (Missall et al., 2015; Zhou et al., 2006). Despite these contrasting findings, a recent systematic review found an overall positive relation between home mathematical practices and children's mathematical ability (Mutaf‐Yıldız et al., 2020), suggesting that it is an important factor to consider when trying to explain how mathematical skills develop.

Currently, less is known about the influence of SES on the frequency of home mathematical activities. A moderate socioeconomic gradient has been found in the home learning environment more broadly (Melhuish et al., 2008). However, our knowledge of whether there are specific socioeconomic gradients in the frequency of home mathematical activities is limited due to the socioeconomically homogeneous samples that are often used in existing research. Of the few studies that attempted to look at this in socioeconomically diverse samples, SES has often been examined in a binary categorical way (medium and low SES; DeFlorio & Beliakoff, 2015; Saxe et al., 1987). This binary approach will be less sensitive to capturing the full influence of SES on home mathematical activities. More recently, Napoli et al. (2021) deployed a more diverse SES sample to explore whether there is an SES gradient in frequency of home mathematical activities. They found a socioeconomic gradient in the frequency of home mathematical activities when age and sex were controlled for. However, the study did not explore whether this SES gradient in home mathematical activities related to variation in early mathematical ability. The importance of using diverse SES samples is further illustrated by home literacy research. SES gradients are clearly apparent in home literacy activities when diverse samples across the full SES spectrum are used (e.g. Phillips & Lonigan, 2009). Therefore, the use of more diverse samples is likely to be useful in elucidating whether there are genuine SES differences in home mathematical activities.

To summarize: previous research identifies four possible factors through which SES attainment gaps in early mathematics may arise—working memory, inhibitory control, verbal ability, and frequency of home mathematical activities. All four factors (i) vary by SES, and (ii) relate to mathematical ability. Moreover, it is vital these factors are considered together, as there are likely to be multiple pathways through which SES influences mathematics. Furthermore, it is most informative to look at these factors before children begin school, as this is when SES disparities first emerge. And it is imperative that low SES groups are included—many prior studies have used predominantly middle‐higher SES samples, greatly limiting what we can learn about socioeconomic attainment gaps. This research is essential in identifying factors that may influence the emergence of these attainment gaps, and will help to inform theoretical models to be tested in longitudinal investigations.

**FIGURE 1:**
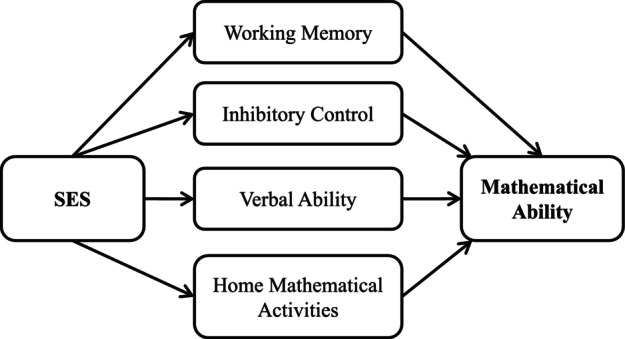
Our hypothesized indirect effects model to explain the relation between socioeconomic status (SES) and mathematical ability.

To that end, the current paper presents two cross‐sectional correlational studies which together aim to identify factors that may explain how SES gaps in early mathematics develop. In both studies, we conduct confirmatory analyses to examine whether working memory, inhibitory control, verbal ability, and home mathematical activities indirectly explain the relation between SES and mathematics, above and beyond general cognitive ability (see Figure 1). Study 2 aims to replicate the novel findings of Study 1, and builds upon Study 1 in three ways. Study 2 recruits a diverse SES sample; it uses a more commonly used measure of frequency of home mathematical activities; and it uses multiple measures of SES, including both a neighborhood‐level and individual‐level measure of SES.

## STUDY 1

The aim of Study 1 was to explore possible factors that may explain the SES gap in early mathematics. Specifically, we examined the extent to which working memory, inhibitory control, verbal ability, and frequency of home mathematical activities indirectly explained socioeconomic gradients in a range of mathematical skills, including counting and cardinality. Counting was included as it is one of the first symbolic mathematical skills to develop (Wynn, 1990). Cardinality was included as it represents the milestone of children mapping number words to meaning (i.e., understanding that the last pronounced number denotes the numerosity of the set) and is vital for subsequent mathematical skills (Geary et al., 2018). A comprehensive standardized index of mathematical ability was also included. To measure frequency of home mathematical activities, this study used a scale by Cahoon et al. (2021) that has recently been developed based on comprehensive parent interviews. To measure SES, the Index of Multiple Deprivation (IMD) was used: this is a precise composite measure of neighborhood‐level SES provided by the UK Office for National Statistics (Ministry of Housing Communities and Local Government, 2019). We predicted that working memory, inhibitory control, verbal ability, and home mathematical activities would vary by SES, and would relate to early mathematical ability (see Figure 1).

### Method

#### Participants

One hundred and seventy‐four children (91 females, 83 males) participated. Children were recruited from six preschools in socioeconomically diverse areas of South Yorkshire, UK in 2019. Data were removed for 15 children (nine children did not complete the tasks due to distraction, three had a language impairment, and three had special educational needs). The final sample comprised 159 children (82 females, 77 males, *M*_age_ = 44 months, SD = 3.95, range = 36–55 months). Sample size was determined through a power calculation to predict mathematical skills from our six predictors and one covariate in a hierarchical regression. The calculation indicated that 158 children would be required to detect a small‐medium effect (*f* ^2^ = .09) with a power of .80 and alpha .05.

**FIGURE 2:**
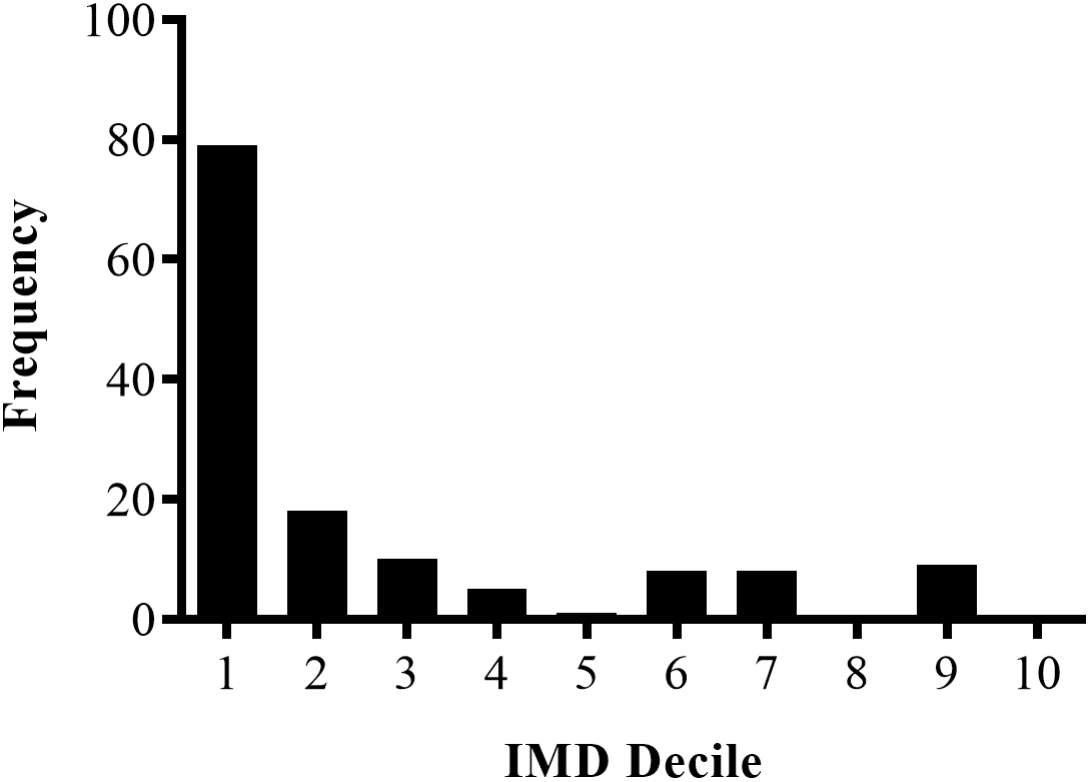
The socioeconomic status distribution of the sample in Study 1 as indexed by the neighborhood deprivation measure Index of Multiple Deprivation (IMD; where 1 represents the most deprived neighborhoods and 10 represents the least deprived neighborhoods).

Parents were asked to complete a questionnaire about the home mathematical environment and their family demographics. Sixty‐nine parents returned questionnaires (43% participation). The final sample of children who had questionnaires returned comprised 33 females and 36 males (*M*_age_ = 44 months, SD = 4.14, range 36–52 months). The ethnicity breakdown for these children was 65% White‐British, 20% Asian, 10% mixed ethnicity, 3% Black‐African, 2% Kurdish. We were able to calculate SES for 87% of the sample (*N* = 138) using the IMD which was gained either from the parent questionnaire or from preschools. IMD scores ranged from 1 (most deprived) to 10 (least deprived). The socioeconomic distribution of the sample, shown in Figure 2, shows that the children in the study are predominantly from lower‐SES households. Returned questionnaires were significantly more likely to be from households in higher‐SES areas, and children with returned questionnaires also had significantly higher scores on the inhibitory control, working memory, verbal ability, counting, cardinality, and test of early mathematics ability (TEMA) tasks. Further details of these comparisons are given in Supporting Information.

#### Measures and procedure

Children were tested individually, completing all seven tasks in a single 45‐min session in their preschool. Tasks were administered in the following fixed order: Give‐a‐Number (cardinality), Black/White Stroop (inhibitory control), Object Span (working memory), Bubble Popping (processing speed), TEMA (standardized mathematical ability measure), Counting, and BPVS (verbal ability). Children were rewarded with stickers for completing the tasks. Following the session, parents were asked to complete the home mathematical activities and family demographic questionnaire.

*Frequency of home mathematical activities* was measured with a questionnaire adapted from Cahoon et al. (2021). Parents rated how frequently they engaged in 26 home mathematical activities with their child—for example, counting objects, playing timed games, or teaching children about money. Frequency was measured using a five‐point Likert scale with the answers ranging from ‘activity did not occur’ to ‘almost daily’ (coded zero to four respectively). Total scores were calculated by adding up the scores for each question (ranging from 0 to 104). The home mathematical activities questionnaire had high reliability (*α* = .89).

*Working memory* was measured with the Object Span task (adapted from Müller et al., 2012). Children were asked to copy a sequence of taps on six familiar objects (*book*, *spoon*, *leaf*, *peg*, *torch*, and *cup*) that were laid out in front of the child. The task contained a short practice phase, followed by three trials at each span length, with spans ranging from one to five (up to 12 trials in total). To progress to the next span length, children had to correctly copy two out of three trials in the correct order. Total scores were calculated by adding up the scores for each correct trial (each correct list position was scored 0.25, with the total score ranging from 0 to 15).

*Inhibitory control* was measured using the Black/White Stroop task (Vendetti et al., 2015). One white card and one black card were placed on a table directly in front of the child. Children were instructed to respond by touching the opposite color card to what they were instructed to do. Therefore, when the experimenter said ‘black’ the child should touch the white card, and when the experimenter said ‘white’ the child should touch the black card. After a short practice phase, children completed 12 trials presented in a fixed pseudorandom order (BA‐BA‐AB‐BA‐BA‐AB). Total scores were calculated by adding up the scores for each correct trial (ranging from 0 to 12). Good test–retest reliability scores for this kind of inhibitory control task have been reported (intraclass correlation coefficient: .87; Lagattuta et al., 2011).

*Verbal Ability* was measured using the British Picture Vocabulary Scale II (BPVS‐II; Dunn et al., 1997). The BPVS is a standardized receptive vocabulary measure normed for children between 3 and 16 years. On each trial, four pictures were presented, and the experimenter read a word aloud. The child was asked to touch the picture that corresponded to the word. The task comprised a short training phase, followed by a testing phase of up to 14 sets of 12 words each, of increasing difficulty. To move onto a higher set, a child would need to give at least 5 correct answers in the current set. Total scores were calculated by adding up the scores for each correct trial (ranging from 0 to 168). The BPVS has been found to have high reliability (*α* = .93; Dunn et al., 1997).

*Counting* was measured using a forward enumeration task where children were asked to count out loud as high as they could, starting from one (up to a maximum of 42). The task was ended if the child gave the wrong number or skipped a number in the count sequence. The total score was the highest number correctly counted to from 1 (ranging from 0 to 42).

*Cardinality* was measured using the Give‐a‐Number task adapted from Wynn (1990). Children were given a basket of 15 toy strawberries, and were told they were the shopkeeper and the experimenter was the customer with an empty basket. The experimenter asked for *n* strawberries to be placed in their basket. *N* followed the order 1, 2, 3, 4, 5, 8, 10. If the child did not place the correct number of strawberries in the basket, the trial was repeated a second time. The task ended if the child did not place the correct number of strawberries in the basket on the second repeated trial. Total scores were calculated by adding up the scores for each correct trial (ranging from 0 to 7).

*Mathematical ability* was assessed using the standardized TEMA‐3 (Form A; Ginsbery & Baroody, 2003). The TEMA‐3 measures a range of early mathematical skills including numeracy, number comparison, numeral literacy, mastery of number facts, calculation skills, and understanding of concepts, in children aged 3–8 years. One point was awarded for each correct answer, and the task ended when five incorrect answers in a row were given. Total scores were calculated by adding up the scores for each correct trial (ranging from 0 to 72). The TEMA‐3 has high reliability and validity (Bliss, 2006).

*Processing speed* was included as a control variable, measured using a computerized ‘bubble‐popping’ task. Children were instructed to ‘pop’ bubbles as fast they could by touching bubbles that appeared on a touchscreen computer (Blakey & Carroll, 2015). Bubbles stayed on the screen until the child had touched them; when children ‘popped’ the bubble, a burst bubble appeared on the screen. Between trials there was an interval varying between 800 and 1200 ms. Children completed a short practice block, followed by eight test trials. Children's median reaction time was calculated.

### Results

#### Descriptive statistics and preliminary analyses

Descriptive statistics and correlation analyses were conducted using SPSS. Mediation analyses were conducted using the PROCESS macro for R (Hayes, 2018) however, we note that because the data are cross‐sectional, we refer to the results as indirect effects, and not mediation (see O'Laughlin et al., 2018 for a discussion). Consequently, results should be interpreted as correlational, and not causal. Data were first examined to check the assumptions for the planned parametric statistical tests. All variables were visually inspected using histograms and P–P plots which revealed that the data for SES, counting, TEMA, and inhibitory control were not normally distributed. Therefore, the non‐parametric correlation Spearman Rho was used to conduct correlation analysis reported in text. Descriptive statistics for the variables of interest, as well as Spearman and Pearson correlation coefficients, are shown in Table 1. The histogram for frequency of home mathematical activities showed substantial variation in the frequency that parents engaged in home mathematical activities with their children, with frequency ranging from 14 to 100 (see Supporting Information). Hierarchical regressions looking at which variables predicted mathematical skills, and alternative mediation models tested can be found in Supporting Information. Age and sex were controlled for in the analyses. This is because prior studies in older children have found executive function mediated the relation between SES and mathematical ability for boys, but not girls (Ellefson et al., 2020).

**TABLE 1:** Spearman's (bottom left) and Pearson's (top right) correlation coefficients for all measures in Study 1 (raw scores).

	*N*	*M* (SD)	1	2	3	4	5	6	7	8	9
1. SES (IMD)	138	2.57 (2.48)		−.04	.25**	.07	−.19*	.35**	.16	.02	.23**
2. Mathematical activities	68	60.01 (16.07)	.02		−.03	−.10	−.08	−.13	.21	−.18	−.07
3. Inhibitory control	155	4.34 (4.21)	.18*	.01		.16*	−.13	.44**	.34**	.17*	.43**
4. Working memory	154	3.51 (1.70)	.12	−.06	.14		−.08	.32**	.30**	.20*	.34**
5. Processing speed	158	1210.92 (277.01)	−.17*	−.05	−.15	−.13		−.21**	−.19*	−.17*	−.29**
6. Verbal ability	157	36.08 (13.49)	.24**	−.17	.42***	.33***	−.19*		.48**	.24**	.51**
7. Cardinality	159	3.55 (1.73)	.12	.18	.34***	.35***	−.17*	.49***		.38**	.62**
8. Counting	149	13.71 (7.29)	.11	−.22	.15	.23**	−.10	.26**	.38***		.60**
9. Mathematical ability	157	6.20 (4.82)	.21*	−.13	.40***	.39***	−.29***	.53***	.59***	.52***	

*Note*: Spearman correlations are displayed in the bottom left corner and Pearson correlations are displayed in the top right corner. *N* = number of participants for each measure. Mathematical Ability refers to the TEMA task.

Abbreviations: IMD, Index of Multiple Deprivation; *M*, mean; SD, standard deviation; SES, socioeconomic status.

* *p* < .05

** *p* < .01

*** *p* < .001.

#### Was there an SES attainment gap in early mathematical ability?

Spearman's correlation analysis revealed a socioeconomic attainment gap in early mathematical ability, with children from more deprived neighborhoods having lower TEMA scores than children from less deprived neighborhoods (*r*_s_(134) = .21, *p* = .015). However, SES did not correlate with counting or cardinality. Given that only the standardized measure of mathematical ability was correlated with SES, this will be the measure of mathematical ability used in the rest of the analyses, and will be referred to as “mathematical ability”.

#### Where do we see SES gradients?

There was a positive correlation between SES and inhibitory control (*r*_s_(132) = .18 *p* = .039), processing speed (*rs*(135)  = −.17, *p* = .044), and verbal ability (*r*_s_(134) = .24, *p* = .005). There were no significant correlations between SES and frequency of home mathematical activities or working memory.

#### Which variables correlated with mathematical ability?

There was a positive correlation between mathematical ability and inhibitory control (*r*_s_(151) = .40, *p* < .001), working memory (*r*_s_(150) = .39, *p* < .001), processing speed (*r*_s_(154) = −.29, *p* < .001), and verbal ability (*r*_s_(153) = .53, *p* < .001). Mathematical ability was not significantly correlated with frequency of home mathematical activities.

#### How can we explain the SES attainment gap in mathematical ability?

Mediation analysis was conducted to explore whether inhibitory control and verbal ability—two factors which both showed a socioeconomic gradient, and both predicted mathematical ability—indirectly predicted the relation between SES and mathematical ability. The first stage of the mediation analysis involved defining the model with direct and indirect effects; the second stage involved assessing the significance of the mediation effects. To assess the significance of our mediation model, we followed Preacher and Hayes's (2008) procedure to calculate the 95% confidence intervals (CIs) of 10,000 bias‐corrected bootstrapping analyses. This was chosen as it is considered a powerful method for detecting an effect while maintaining control over Type 1 errors, making it superior to other mediation procedures such as the Sobel test. A significant mediated effect is indicated if the CIs do not pass through zero.

**FIGURE 3:**
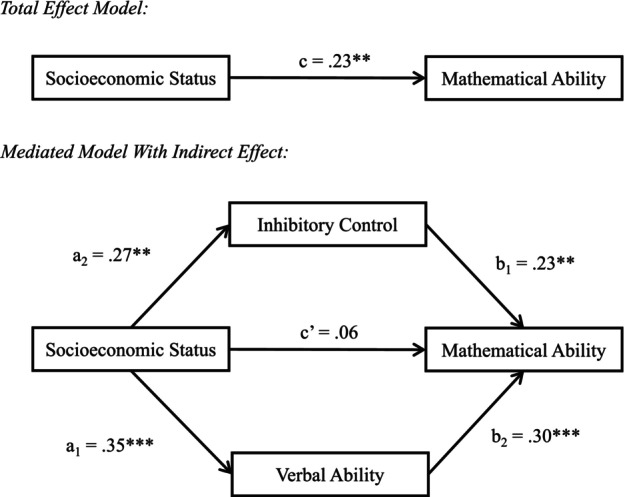
Mediation model showing the relation between socioeconomic status Index of Multiple Deprivation (IMD) and test of early mathematics ability (TEMA) is indirectly explained by inhibitory control and verbal ability, controlling for processing speed, age and sex. Standardized beta weights are given. *p < .05, **p < .01, ***p < .001.

In order to test whether inhibitory control and verbal ability indirectly explained the relation between SES and mathematical ability, a mediation analysis with two indirect effects was conducted. The model was fit with SES as the predictor; inhibitory control and verbal ability as mediators; and mathematical ability as the outcome variable. Processing speed, age, and sex were included as covariates (Figure 3). In the total effect model, SES had a significant positive effect on mathematical ability (*β* = .23, *p* = .006). In the mediated model, SES had a significant positive effect on inhibitory control (*β* = .27, *p* = .002) and verbal ability (*β* = .35, *p* < .001). Inhibitory control (*β* = .23, *p* = .009) and verbal ability (*β* = .30, *p* < .001) had significant positive effects on mathematical ability. The results of the bootstrapping procedure revealed that the indirect effect through inhibitory control (95% CI [0.02, 0.14]) and the indirect effect through verbal ability (95% CI [0.03, 0.21]) were significant, as the CIs did not pass through zero. The CIs indicated that inhibitory control and verbal ability were significant indirect effects in the relation between SES and mathematical ability. Pairwise contrasts of the indirect effects through inhibitory control and verbal ability (95% CI [−0.07, 0.16]) indicated that the paths did not differ significantly from each other, as the CIs passed through zero.

### Discussion

The aim of Study 1 was to explore whether working memory, inhibitory control, verbal ability, and frequency of home mathematical activities are factors that may influence SES disparities in mathematical ability in the preschool years. Three measures of mathematical skill were assessed in the study: a standardized measure of general mathematical ability, and two measures of specific mathematical skills: counting and cardinality. The study found a socioeconomic attainment gap in the standardized measure of mathematical ability, but no socioeconomic gradients were found in counting and cardinality. Frequency of home mathematical activities did not vary by SES, and did not relate to any measure of mathematical ability. In contrast, working memory, inhibitory control, and verbal ability all positively correlated with mathematical ability—but socioeconomic gradients were only found in inhibitory control and verbal ability. As inhibitory control and verbal ability both correlated with mathematical ability and SES, we examined the extent to which they indirectly explained early attainment gaps in mathematical ability using mediation analyses. Both inhibitory control and verbal ability emerged as indirect predictors in the relation between SES and mathematical ability. This is some of the first research to show that verbal ability and inhibitory control may be important factors to explain how mathematical attainment gaps arise.

Contrary to our hypotheses, neither working memory nor home mathematical activities varied by SES, suggesting these are not mechanisms by which SES attainment gaps in early mathematical ability develop. With regards to working memory, the fact that no socioeconomic gradient was found is at odds with some previous research (Lawson et al., 2018). One potential explanation for this difference is the age of the children tested. The majority of studies looking at SES gradients in working memory have been conducted with school‐age children. Our study looked at preschoolers, and it is conceivable that SES gradients in working memory only emerge later in development. With regards to home mathematical activities, less is known about the role of SES, meaning that it is quite possible that the results of this study finding no SES gradient hold true. However, the lack of relation between SES and both working memory and home mathematical activities requires further exploration before these conclusions can be accepted with confidence. There are two reasons for this. Firstly, the sample in the present study was drawn from predominantly low‐SES neighborhoods. In order to be confident there are no SES gradients in working memory and home mathematical activities, a more diverse sample from across the full SES spectrum would be needed, to ensure enough variation to be able to detect possible differences. Secondly, the present study used neighborhood indices of SES, rather than individual indices. Previous research into working memory found SES differences when using parent education, but not when using a neighborhood deprivation measure (Hackman et al., 2014). While the IMD has been found to strongly relate to educational outcomes (Crawford & Greaves, 2013), it reflects the average SES for a neighborhood. Therefore, using individual measures of SES (such as parental education) would give a more accurate measure of an individual child's SES.

The absence of relation between home mathematical activities and mathematical ability is particularly interesting, as many previous studies have reported this relation (see Mutaf‐Yıldız et al., 2020 for review). More broadly, it seems to go against the notion that practicing a skill will lead to improvements in that skill. There are three further reasons to be cautious about this null finding. Firstly, it is important to note that while the current study was well powered overall, there was a low questionnaire return rate (43%). This meant that the study only had 51% power to detect a small‐medium effect with home mathematical activities—although interestingly, the non‐significant correlation between home mathematical activities and mathematical ability was small and *negative*. Secondly, Study 1's mostly low‐SES sample may have meant there was little variation in home mathematical activities. Furthermore, higher‐SES families were more likely to return the home mathematical activities questionnaire than lower‐SES families, limiting the conclusions that can be drawn about the relation between SES and home mathematical activities. Thirdly, Study 1 used a new measure of home mathematical activities (Cahoon et al., 2021), while the majority of previous studies in this area have used a questionnaire based on the one developed by Lefevre et al. (2009). It may be that the questions developed by Lefevre et al. (2009) better capture the home mathematical activities relating to mathematical ability. So, while Study 1 is not the first study to find no link between home mathematical activities and mathematical ability (see also Missall et al., 2015; Zhou et al., 2006), there are grounds to be cautious when interpreting this null result.

In summary, the results of Study 1 suggest two possible factors that may explain how early attainment gaps in mathematical skills emerge—verbal ability and inhibitory control. Furthermore, the results indicate that working memory and home mathematical activities may not be factors that influence early attainment gaps in mathematics. This would be an important finding that sheds new light on early‐developing attainment gaps. However, given the relative novelty of these findings, it is important to test their robustness. Thus, we aimed to extend and replicate these findings in a further study.

## STUDY 2

The aim of Study 2 was to replicate the findings of Study 1. Like Study 1, Study 2 explores the role of working memory, inhibitory control, verbal ability, and frequency of home mathematical activities as mechanisms for explaining the SES attainment gap in early mathematical ability. However, in Study 2 we made three methodological changes to ensure we could be confident in the results of Study 1. Firstly, the study aimed to recruit a socioeconomically diverse sample, to ensure we were capturing full variation across the SES spectrum. Secondly, the study took an individual measure of SES (mother's education) in addition to a neighborhood measure. Thirdly, the study used a different measure of home mathematical activities, which has been used more widely in the existing literature. To measure mathematical ability, we retained only the standardized measure of mathematical ability, since Study 1 only found a SES gradient on this broader and more comprehensive measure. All other measures remained the same.

### Method

#### Participants

One hundred and forty‐five preschoolers (80 females, 65 males, *M*_age_ = 45.38 months, SD = 4.13, range = 37–52 months) participated between 2018 and 2019. Of these, 113 preschoolers were recruited from five preschools in socioeconomically diverse areas of South Yorkshire, UK, and 32 children were recruited from a database of local families who had expressed an interest in participating in research. Sample size was determined through a power calculation to predict mathematical skills from five predictors and one covariate in a hierarchical regression. This indicated that 149 children were required to detect a small‐medium effect (*f* ^2^ = .09) with a power of .80 and alpha .05.

Parents were asked to complete a questionnaire which collected data on demographic information, SES, and frequency of home mathematical activities. One hundred and six parents returned the questionnaire (73% participation, an increase from Study 1). The final sample of preschoolers who had questionnaires returned comprised 57 females and 49 males, *M*_age_ = 45 months, SD = 4.10, range 37–52 months. The ethnicity breakdown for the children was 86% White‐British, 9% White‐other background, 4% Asian, 1% mixed ethnicity. Comparing the preschoolers whose parents completed the questionnaire to those who did not indicated no differences on the experimental tasks. It was not possible to assess SES differences in questionnaire return, as we only had SES information for children who returned the questionnaire. Further details of these comparisons are given in Supporting Information.

**FIGURE 4:**
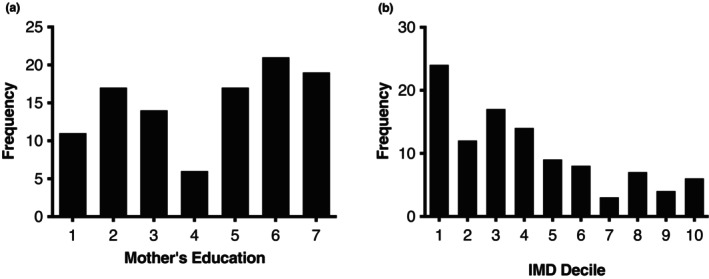
The socioeconomic status (SES) distribution of the sample in Study 2 as indexed by: (a) mother's education (where 1 is the lowest and 7 is the highest level of education) and (b) the neighborhood deprivation measure Index of Multiple Deprivation (IMD) (where 1 is the most deprived and 10 is the least deprived).

Socioeconomic status was calculated for each child using two measures: IMD and mother's highest level of education. IMD was derived from household postcode; for mother's education, the questionnaire asked for their highest level of education from a set list, ranging from ‘no formal qualifications’ to ‘postgraduate degree or similar’. The European Qualification Framework (European Commission, 2018) was used to score the qualification, which ranged from 1 (lowest level of education) to 7 (highest level of education). The socioeconomic distribution of the sample, displayed in Figure 4, showed that the sample was socioeconomically diverse. In this study we focus on the more direct measure of SES, parent education (further details below).

#### Measures and procedure

Children completed all five tasks in a single session, either in their preschool or the university laboratory. Tasks were administered in a fixed order: Black/White Stroop (inhibitory control), TEMA (mathematical ability), Bubble Popping (processing speed), Object Span (working memory), and BPVS (verbal ability). Testing lasted approximately 40 min, and children were rewarded with stickers for completing the tasks. In addition, parents were asked to complete the questionnaire on home mathematical activities and family demographics.

Measures were identical to those in Study 1, with the single exception that frequency of home mathematical activities was measured using an alternative parent questionnaire. Parents were asked to rate how frequently they engaged in 21 mathematical activities with their child—for example, writing numbers, using calendars and dates, playing board games with a die or spinner (questions adapted from Lefevre et al., 2009). Frequency was measured using a five‐point Likert scale, with the answers ranging from ‘activity did not occur’ to ‘activity occurred almost daily’ (coded 0–4 respectively). Total scores were calculated by adding up the scores for each question (ranging from 0 to 84). The home mathematical activities questionnaire had high reliability (*α* = .89).

### Results

#### Descriptive statistics and preliminary analyses

Analyses were run in the same way as for Study 1. First, all variables were visually inspected using histograms and P–P plots which revealed that the data for mathematical ability, working memory, and inhibitory control were not normally distributed. Therefore, the non‐parametric correlation Spearman Rho was used to conduct correlation analysis reported in the text. Descriptive statistics for the variables of interest, as well as Spearman and Pearson correlation coefficients, can be seen in Table 2. The histogram for frequency of home mathematical activities showed substantial variation in the frequency that parents are engaging in home mathematical activities with their children, with frequency ranging from 4 to 73 (see Supporting Information). Hierarchical regressions looking at which variables predicted mathematical ability, and alternative mediation models tested can be found in Supporting Information. Age and sex were controlled for in the regression and mediation analyses.

**TABLE 2:** Spearman's (bottom left) and Pearson's (top right) correlation coefficients for all measures in Study 2 (raw scores).

	*N*	*M* (SD)	1	2	3	4	5	6	7	8
1. SES (IMD)	104	4.05 (2.75)		.48**	−.03	−.17	.28**	.07	.37**	.28**
2. SES (mother education)	105	4.33 (2.06)	.48***		−.03	−.21*	.25**	.16	.40**	.30**
3. Mathematical activities	105	32.32 (13.27)	.00	−.07		−.05	.04	.14	−.06	.12
4. Processing speed	142	1306.10 (338.52)	−.21*	−.24**	.03		−.17*	−.13	−.29**	−.29**
5. Inhibitory control	145	5.83 (4.18)	.30**	.26**	.06	−.16		.16*	.34**	.50**
6. Working memory	145	2.90 (2.01)	.07	.17	.15	−.14	.17*		.33**	.49**
7. Verbal ability	144	37.87 (13.30)	.35***	.39***	−.04	−.28**	.36***	.33***		.55**
8. Mathematical ability	142	6.72 (6.03)	.33**	.40***	.12	−.29**	.48***	.48***	.62***	

*Note*: Spearman correlations are displayed in the bottom left corner and Pearson correlations are displayed in the top right corner. *N* = number of participants for each measure.

Abbreviations: IMD, Index of Multiple Deprivation; *M*, mean; SD, standard deviation; SES, socioeconomic status.

* *p* < .05

** *p* < .01

*** *p* < .001.

#### Is there an SES attainment gap in early mathematical ability?

Spearman's correlation analysis revealed a socioeconomic attainment gap in early mathematical ability, as indexed by both IMD and mother's education. Lower levels of mathematical ability were evident in children living in more deprived neighborhoods (*r*_s__(100)_ = .33, *p* = .001), and in children whose mothers had a lower level of education (*r*_s__(101)_ = .40, *p* < .001). For all further analyses, we used mother's education as the primary measure of SES, as this provides the most direct measure of family SES.

#### Where do we see SES gradients?

There were significant positive correlations between SES and inhibitory control (*r*_s__(103)_ = .26, *p* = .005), and SES and verbal ability (*r*_s__(102)_ = .39, *p* < .001). SES was not significantly correlated with either working memory or frequency of home mathematical activities.

#### Which factors correlated with mathematical ability?

Mathematical ability was significantly positively correlated with inhibitory control (*r*_s__(141)_ = .48, *p* < .001), working memory (*r*_s_(141) = .48, *p* < .001) and verbal ability (*r*_s_(140) = .62, *p* < .001). It was not significantly correlated with frequency of home mathematical activities, indicating that frequency of home mathematical activities does not influence early mathematical ability.

#### How can we explain the SES attainment gap in mathematical ability?

**FIGURE 5:**
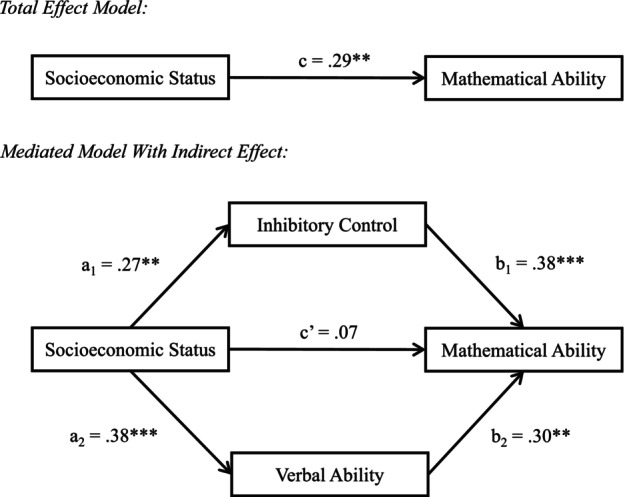
Mediation model showing the relation between socioeconomic status (mother's education) and mathematical ability is indirectly explained by inhibitory control and verbal ability, controlling for processing speed, age, and sex. Standardized beta weights are given. *p < .05, **p < .01, ***p < .001.

To test whether inhibitory control and verbal ability mediated the relation between SES and mathematical ability, a mediation analysis with two indirect effects was conducted. The model was fit with SES as the predictor; inhibitory control and verbal ability as indirect effects; and mathematical ability as the outcome variable. Processing speed, age, and sex were included as covariates (Figure 5). In the total effect model, SES had a significant positive effect on mathematical ability (*β* = .29, *p* = .003). In the mediated model, SES had a significant positive effect on inhibitory control (*β* = .27, *p* = .009) and verbal ability (*β* = .38, *p* < .001). Inhibitory control (*β* = .38, *p* < .001) and verbal ability (*β* = .30, *p* = .001) had significant positive effects on mathematical ability. The results of the bootstrapping procedure revealed that the indirect effect through inhibitory control (95% CI [0.03, 0.20]) and the indirect effect through verbal ability (95% CI [0.04, 0.22]) were significant, as the CIs did not pass through zero. The CIs indicated that inhibitory control and verbal ability mediate the relation between SES and mathematical ability. Pairwise contrasts of the indirect effects through inhibitory control and verbal ability (95% CI [−0.12, 0.14]) indicated that the paths did not differ significantly from each other, as the CIs passed through zero.

### Discussion

Study 2 aimed to replicate the findings of Study 1 using a more direct measure of SES, in a very diverse sample, and using a different scale to measure home mathematical activities. The results of Study 1 were fully replicated: inhibitory control and verbal ability explained the relation between SES and mathematical ability; working memory significantly related to mathematical ability, but was not found to vary by SES; and the frequency of home mathematical activities did not vary by SES, or relate to frequency of mathematical ability. The replication of these findings suggests we can be more confident in our conclusions: that inhibitory control and verbal ability are possible factors by which SES attainment gaps in early mathematics emerge; but working memory and frequency of home mathematical activities are not. However, it is important to note that due to the cross‐sectional nature of the data, these results are indicative, and not confirmatory. The results should be interpreted as a way to inform future longitudinal research establishing causal inference, as a first step to establishing possible interventions. We now discuss these findings in more detail.

## GENERAL DISCUSSION

The present research aimed to explore possible factors that influence early SES gaps in mathematical ability. Two studies were conducted to explore four possible factors: working memory, inhibitory control, verbal ability, and frequency of home mathematical activities. These factors were chosen because previous studies—which tend to look at them in isolation—found that they show SES gradients, and support children's mathematical skills. Study 1 explored the role of these factors in explaining SES attainment gaps (as indexed by neighborhood deprivation) on three measures of mathematical ability. Study 2 replicated and extended this work, by using an individual measure of SES, and recruiting a highly socioeconomically diverse sample. Together, these studies provide a comprehensive exploration of factors that may influence SES gaps in mathematical ability in early childhood. Crucially, both studies show that verbal ability and inhibitory control may be key to explaining early attainment gaps.

Four key findings emerged from this research. Firstly, there was a clear SES attainment gap in early mathematical ability. Secondly, verbal ability indirectly predicted the relation between SES and early mathematical ability. Thirdly, inhibitory control indirectly predicted the relation between SES and mathematical ability. Fourthly, working memory and home mathematical activities did *not* explain SES disparities in mathematical ability in either study. The fact that both studies—with different samples and different measures of SES and home mathematical activities—align on all four key findings suggests these results are robust. We now discuss each of these findings in more detail.

The first key finding was that a SES attainment gap in mathematical ability is apparent in children as young as 3 years of age. This is striking, and demonstrates that there are factors at play *before* children start school that lead to inequalities in outcomes. We know that mathematical development proceeds cumulatively (Baroody et al., 2012), meaning this early attainment gap is likely to not only remain, but to widen over time. This underlines the importance of targeting attempts to narrow this gap to the preschool years. The fact that SES gradients were not seen in basic measures of mathematical skills—counting and cardinality—is interesting, and worthy of further study. It may indicate that SES does not affect *all* kinds of mathematical skills equally. One explanation is that counting and cardinality are very basic tasks which children may complete without a real understanding of numerical magnitude (Sella & Lucangeli, 2020). In contrast, our measure of overall mathematical ability (the TEMA) required children to complete more complex tasks, and to use skills in combination (e.g., magnitude comparison and the use of arithmetic facts). Therefore, the mechanisms leading to SES gradients in mathematical skills may be ones that affect children's ability to integrate and use mathematical skills in concert. This is entirely consistent with the idea that SES may correlate with mathematical skills due to differences in children's executive functions (see Blakey et al., 2020). It would therefore follow that the biggest SES gradients on mathematical tasks would be seen on tasks that require children to use multiple mathematical skills in parallel.

The second key finding was that SES attainment gaps in early mathematics were indirectly explained by verbal ability. This is a key finding that bridges two important areas of research. Firstly, the finding that there are SES differences in verbal ability is consistent with a wealth of research demonstrating SES gradients in verbal ability (e.g. Fernald et al., 2013). Secondly, the findings are also consistent with a separate, growing body of evidence demonstrating the importance of verbal ability in the development of mathematical skills (Purpura et al., 2011; von Stumm et al., 2020). Verbal ability may support mathematical skills in multiple ways. Notably, language is essential for attributing meaning to arbitrary mathematical concepts, and for expressing those meanings. It is also conceivable that verbal ability may modulate the cognitive demands of a task: for example, a child with poor verbal ability may not only have to meet the demands of the mathematical task itself, but also of learning, understanding and using unfamiliar language when completing the task (Meyer, 2000). By connecting these two separate strands of research—one examining SES gradients in verbal ability, and one on verbal ability and mathematical skills—the current study provides a key factor for longitudinal research to explore. If longitudinal evidence does indicate causation, this would be promising, as there are already a number of verbal ability interventions which have been found to be effective (Marulis & Neuman, 2010).

The third key finding was that SES attainment gaps in early mathematics were also indirectly explained by inhibitory control. This finding is consistent both with previous research showing inhibitory control to be important for early mathematical development (Allan et al., 2014), and with studies that have found SES gradients in inhibitory control (Blakey et al., 2020; Lawson et al., 2018). To date, little is known about the mechanisms by which SES influences the development of inhibitory control. Recent longitudinal research may shed light on this. Waters et al. (2021) found that inhibitory control mediates the relation between SES and later mathematical ability, but that this relation did not hold when controlling for verbal ability. This finding suggests that verbal ability may be important in explaining SES differences in inhibitory control. Indeed, there is some evidence to suggest that verbal ability may help scaffold executive function development, by enabling children to monitor their thoughts and actions using inner speech (Daneri et al., 2019). In line with these findings and the current findings, we speculate that verbal ability may be a critical mechanism that explains how disparities in children's mathematical skills arise through its impact on executive function skills. Specifically, SES disparities in mathematical skills may begin by SES influencing early verbal ability; this, in turn, may have a knock‐on‐effect on inhibitory control; which then goes on to influence mathematical ability. This speculation may inform a finer‐grained model of how SES attainment gaps emerge, though it would be for future longitudinal research to definitively test such a model.

The fourth key finding was that SES attainment gaps in early mathematics were not explained by working memory or by home mathematical activities. Neither factor showed SES gradients, and thus neither could explain attainment gaps in mathematical ability. Both studies showed that working memory was positively related to mathematical ability, but that working memory did not vary by SES. This suggests that despite working memory's importance for early mathematical skills, it may not be a mechanism that drives SES disparities in mathematics. While the results were consistent across studies one and two, we would nevertheless suggest that working memory should not be entirely dismissed as a possible mechanism by which SES gaps could emerge—and that the way one operationalizes working memory may be crucial. In our studies, the working memory measure mostly indexed young children's visuospatial recall skills; this contrasts with the more complex working memory measures typically used with older children, which index the ability to manipulate and update information. Indeed, previous research has found small‐medium SES differences in older children's working memory, using a working memory task with a manipulation element (Blakey et al., 2020; Lawson et al., 2018). Recent work has also identified working memory as a mediator of attainment gaps when a verbal measure is used (Waters et al., 2021). Therefore, it is possible that SES differences in the ability to process and manipulate information emerge over time and are more likely in the verbal domain, perhaps because SES shows gradients in language ability.

Somewhat unexpectedly, both Studies 1 and 2 found that home mathematical activities did *not* relate to children's mathematical ability—and nor did they vary by SES. Therefore, SES gaps in early mathematical ability do not appear to arise as a function of differences in frequency of home mathematical activities. The absence of relation between frequency of home mathematical activities and mathematical ability is surprising, and worth further attention, not least because prior research on this topic has yielded contrasting results. The present findings are consistent with some prior studies (Missall et al., 2015; Zhou et al., 2006), but not others (Kleemans et al., 2012; Lefevre et al., 2009). One possible explanation could be the age of children: the current study was conducted with children prior to the start of formal education, in contrast to Lefevre et al.'s (2009) seminal study, which was conducted after children had begun formal education. It may be that the relation between home mathematical activities and mathematical ability is age‐specific, with it emerging as children get older and are able to do more complex mathematical operations (Thompson et al., 2017). Another possible explanation for the diversity in findings might be the socioeconomic features of the samples tested. The majority of research into the frequency of home mathematical activities has been conducted with socioeconomically homogeneous samples. It is possible that the relation between frequency of home mathematical activities and mathematical abilities does not have the same strength across the SES spectrum, and is perhaps seen most strongly in higher‐SES families. This would go some way towards explaining why no relation was found in Study 1 (with a predominantly lower‐SES sample) or in Study 2 (with a diverse SES sample). This suggestion is wholly consistent with a meta‐analysis showing that the positive relation between home mathematical activities and mathematical ability was larger in high‐SES families than low‐SES families (Dunst et al., 2017). The fact that both the present studies show this null relation, and that both use different home mathematical activities questionnaires, gives us confidence that in socioeconomically diverse samples, *frequency* of home mathematical activities does not influence mathematical ability prior to the start of formal education.

The absence of a SES gradient in frequency of home mathematical activities is noteworthy in its own right. Little previous research has directly explored this topic, and the few studies that do have tended to use socioeconomically homogeneous samples, or relatively basic measures of SES. The present research featured two commonly used measures of SES, and a diverse SES sample, and still found no relation between SES and frequency of home mathematical activities. This indicates that SES really does not appear to influence the frequency in which parents engage in home mathematical activities with their children.

While this research did not find *frequency* of home mathematical activities to be a mechanism by which SES attainment gaps emerge, it is nevertheless conceivable that there is still a role to be played by home mathematical activities. It may be that a different picture will emerge when one considers not simply the *frequency* of mathematical activities in the home, but rather the *type*, *range* and *quality* of those activities. Indeed, language research has shown the importance of quality over quantity for a child's verbal ability (Hirsh‐Pasek et al., 2015). It remains a possibility that type, range and quality of mathematical activities may influence mathematical development, and may themselves be differently influenced by SES. To test this possibility, studies need to go beyond questionnaire scales in order to gather richer data—for example, by using interviews and observations in the home, to fully capture the diversity of interactions and activities that may have mathematical components embedded (see Elliott et al., 2020). This suggestion is supported by research comparing questionnaire data with semi‐structured interview data on home mathematical activities which found they did not correlate with one another (Mutaf Yıldız et al., 2018). Alternatively, it may be that the frequency of home mathematical activities predicts *growth* in mathematical ability, rather than a child's ability at a single time point, with early activities potentially providing a foundation to support the future acquisition of mathematical skills. Better understanding how the type, range and quality of home mathematical abilities contribute to mathematical skills over time will be an important avenue for future research.

It is important to note limitations of the studies presented. The first and most important limitation concerns their cross‐sectional nature, meaning it is not possible to disentangle any causal direction among variables from the cross‐sectional correlational data. While we did test plausible alternate models (see Supporting Information) and they explain less variance than our hypothesized models, the nature of the correlational data means that these alternate mediation models also provide statistically plausible indirect effects. Thus, while the current studies do provide a clear and robust account of theoretically grounded factors that may be of interest, longitudinal research is needed to confirm the temporal ordering of these variables before conclusions can be drawn about causal mechanisms. Therefore, the results of the current study should be interpreted as descriptive and used to inform future longitudinal work rather than seen as any recommendation for applied interventions. The identification of temporal ordering prior to intervention is crucial as a bidirectional relation between the development of the executive functions and mathematical ability has been speculated (Schmitt et al., 2017). However, we note that a recent paper failed to replicate this finding in two large samples indicating that the causal relation does go executive functions to mathematical ability, not vice versa (Ellis et al., 2021). Existing longitudinal research has been particularly helpful in identifying predictors of later mathematical achievement. They have been valuable in demonstrating the importance of SES and early executive functions on later skills (Ahmed et al., 2018; Waters et al., 2021). However, of the longitudinal studies on this topic, many focus on older children who have started formal schooling, or do not look at mediators of attainment gaps directly. Instead, they elucidate predictors of later achievement and have extensive control variables (including language and SES; Ahmed et al., 2018); or when they do examine attainment gaps, focus on executive functions (Waters et al., 2021). The predictors of attainment gaps, as this work has identified, are likely to be multi‐factorial so it will be helpful for future work to examine how multiple predictors like executive functions, language, *and* the quality of activities in the home predict mathematical attainment gaps as they emerge early on and change over time.

Another limitation relates to the measurement of some of our variables. Firstly, our measure of working memory may have relied more on short‐term memory and that may be why we found little relation with working memory and SES. As yet, there are no sensitive working memory tasks for 3‐year‐olds that require both storage and processing. It would be beneficial for future research to develop such measures. Secondly, measuring the frequency of home mathematical activities may not capture the full range of activities parents do with their children, nor the quality of such activities. It would be helpful for future research to move beyond questionnaires, to measures that can capture the quality and breadth of home activities such as through observation. Thirdly, we focused on measuring factors that were most proximal to mathematical development. However, other more distal factors may also be important in explaining SES attainment gaps, including mathematical vocabulary. Mathematical vocabulary has been found to relate to preschoolers' early mathematical skills (King & Purpura, 2021), but less is known about its relation to SES. It would be fruitful to explore whether there is an SES gradient in children's mathematical vocabulary—and if so, whether mathematical vocabulary is a mediating factor to explain SES attainment gaps.

The present research is the first to directly investigate the specific factors that may explain SES gaps in early ability in a diverse socioeconomic sample. In two studies, we find that SES attainment gaps for mathematical ability are explained by both verbal ability and inhibitory control. We find that frequency of home mathematical activities does not vary by SES or influence early mathematical ability and it may be fruitful for future work to focus on the *quality* of these activities above and beyond frequency. By examining multiple factors, these studies indicate how early SES gaps in mathematical ability may arise. The findings offer a vital first step towards designing longitudinal studies to elucidate on the causal sequence and long‐term consequences of these early inequalities.

## Supporting information


Appendix S1.

## Data Availability

Anonymised data from this study is available on the Open Science Framework here https://osf.io/buphv/?view_only=4ec18e3ae6584ae0b77badbb408a9514. Tasks scripts and protocols are available from the corresponding author.
