# Supplementary material for: How do socioeconomic attainment gaps in early mathematical ability arise?
Source: Child Dev. 2023 May 30;94(6):1550–65. doi: 10.1111/cdev.13947 (PMC10953023; doi:10.1111/cdev.13947)
Supplement: Supplementary file 1 — Appendix S1. [file CDEV-94-1550-s001.docx]

Did children who returned the home mathematical questionnaire differ from children who did not return the home mathematical questionnaire?

To examine whether children who had a questionnaire returned compared to those who did not significantly differed on task performance, an independent samples t-test was conducted. To deal with variables that violated the assumption of normality, confidence intervals with bias corrected accelerated bootstrapped are reported. The results of the t-test for study one are displayed in Table 1, and the results of the t-test for study two are displayed in Table 2.

Table S1. Task scores for children who had returned a questionnaire compared to those who did not have a returned questionnaire for study one.

| **Study One** | **Questionnaire Returned** | |  | | **Questionnaire not returned** | | **Mean difference** | ***t (df)*** | ***p* value** | **95% CI** | |
| --- | --- | --- | --- | --- | --- | --- | --- | --- | --- | --- | --- |
|  | ***M*** | ***SD*** |  | ***M*** | | ***SD*** |  |  |  |  |  |
| **SES (IMD)** | 3.68 | 2.94 |  | 1.46 | | 1.12 | 2.22 | 5.86 (87) | .001 | 1.58, 2.84 |  |
| **Inhibitory Control** | 5.23 | 4.38 |  | 3.62 | | 3.74 | 1.62 | 2.39 (153) | .014 | 0.26, 2.88 |  |
| **Working Memory** | 3.95 | 1.88 |  | 3.17 | | 1.46 | 0.78 | 2.90 (152) | .004 | 0.26, 1.35 |  |
| **Verbal Ability** | 40.04 | 13.62 |  | 33.12 | | 12.68 | 6.92 | 3.28 (155) | .003 | 2.91, 10.67 |  |
| **Processing Speed** | 1211.88 | 264.63 |  | 1210.19 | | 287.72 | 1.69 | 0.38 (156) | .971 | -76.60, 88.19 |  |
| **Counting** | 15.35 | 7.84 |  | 12.51 | | 6.64 | 2.84 | 2.33 (120) | .023 | 0.48, 5.39 |  |
| **Cardinality** | 3.93 | 1.95 |  | 3.27 | | 1.48 | 0.66 | 2.34 (123) | .019 | 0.10, 0.17 |  |
| **Mathematical Ability** | 7.66 | 5.31 |  | 5.08 | | 4.10 | 2.58 | 3.32 (123) | .004 | 1.13, 4.09 |  |

*Note. M* = Mean, *SD* = Standard Deviation, *df* = degrees of freedom, *CI* = Confidence Intervals reported with bias corrected accelerated (BCa) bootstrapping based on 1000 samples, Mathematical Ability refers to the TEMA task.

Table S2. Task scores for children who had returned a questionnaire compared to those who did not have a returned questionnaire for study two.

| **Study Two** | **Questionnaire Returned** | |  | | **Questionnaire not returned** | | **Mean difference** | ***t (df)*** | ***p* value** | **95% CI** |
| --- | --- | --- | --- | --- | --- | --- | --- | --- | --- | --- |
|  | ***M*** | ***SD*** |  | ***M*** | | ***SD*** |  |  |  |  |
| **Inhibitory Control** | 6.09 | 4.05 |  | 5.10 | | 4.49 | 0.99 | 1.27 (143) | .214 | -0.90, 2.81 |
| **Working Memory** | 3.05 | 2.04 |  | 2.51 | | 1.86 | 0.53 | 1.43 (143) | .138 | -0.18, 1.19 |
| **Verbal Ability** | 38.55 | 13.83 |  | 36.05 | | 11.71 | 2.50 | 1.00 (142) | .293 | -2.23, 7.05 |
| **Processing Speed** | 1403.57 | 362.85 |  | 1485.63 | | 368.71 | -82.07 | 1.19 (140) | .204 | -227.64, 62.71 |
| **Mathematical Ability** | 7.12 | 6.30 |  | 5.67 | | 5.14 | 1.45 | 1.28 (141) | .166 | -0.61, 3.43 |

*Note. M* = Mean, *SD* = Standard Deviation, *df* = degrees of freedom, *CI* = Confidence Intervals reported with bias corrected accelerated (BCa) bootstrapping based on 1000 samples.

Is there variation in frequency of home mathematical activities?

To explore whether there is variation in the frequency that parents engage in home mathematical activities with their child, histograms were visually inspected. Figure X displays the frequency for study one, and Figure X displays the frequency for study two.


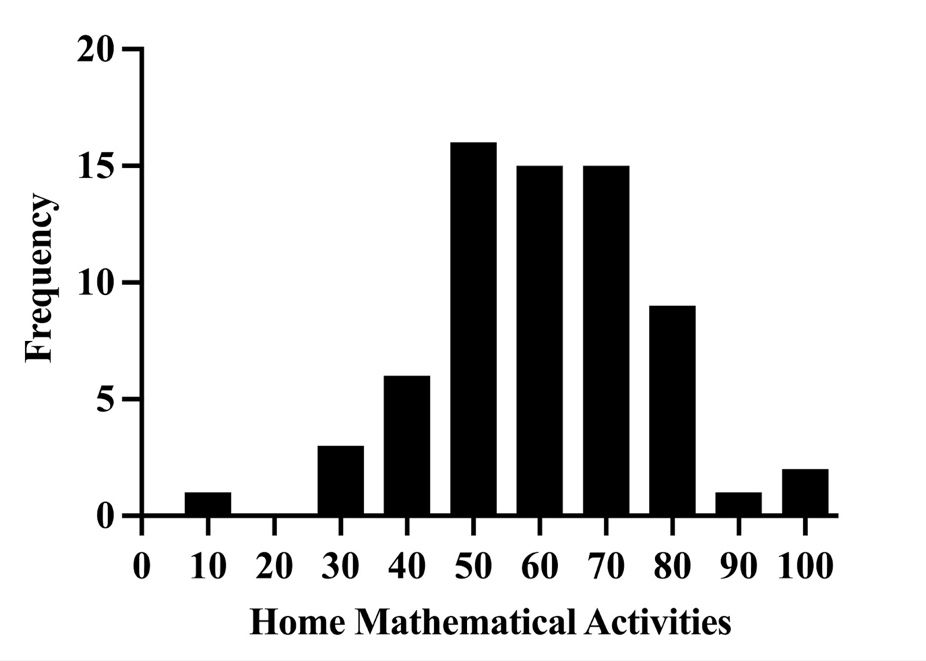


**Figure S1** displays the frequency that parents engaged in home mathematical activities with their child for Study One.


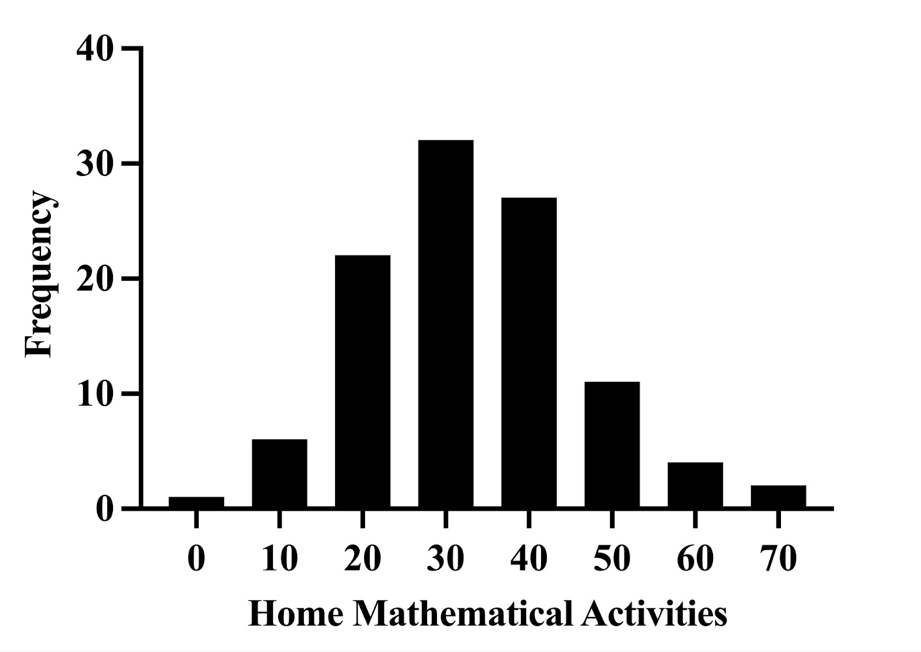


**Figure S2** displays the frequency that parents engaged in home mathematical activities with their child for Study Two.

Both histograms reveal substantial variation in the frequency that parents are engaging in home mathematical activities with their child.

Hierarchical regression predicting mathematical ability from our predictors

To examine which variables predicted early mathematical ability, for both study one and study two data, a three-model hierarchical multiple regression was conducted that included variables that significantly correlated with mathematical ability.

Assumptions for regression analysis were checked. For study one, the assumptions of linearity, multicollinearity and homoscedasticity were met, but the assumption of normality of residuals was violated. In order to meet the assumption of normality of residuals, a log_10_+1 transformation was applied to the dependent variable mathematical ability (TEMA). For study two, all assumptions were met.

For both studies, age, sex, and processing speed were included as a covariate for in all models. In Model 1, SES was entered. In Model 2, inhibitory control and working memory were entered. In Model 3, verbal ability was entered. The statistics for the study one analysis are reported in Table 3 and the statistics for study two analysis are reported in Table 4.

**Table S3**. Results of the Hierarchical Regression Analysis Predicting mathematical ability for Study One.

|  | **Predictor** | ***b***  ***(95% confidence intervals)*** | ***SE B*** | ***β*** | ***p*** |
| --- | --- | --- | --- | --- | --- |
| **Model 1** | Constant | -0.52  (-1.20, 0.16) | 0.34 |  | .135 |
|  | SES (IMD) | 0.03  (0.01, 0.05) | 0.01 | .21 | .009 |
|  | Sex | 0.03  (-0.07, 0.13) | 0.05 | .05 | .550 |
|  | Age | 0.03  (0.02, 0.05) | 0.01 | .38 | < .001 |
|  | Processing Speed | 0.00  (0.00, 0.00) | 0.00 | -.15 | .068 |
| **Model 2** | Constant | -0.22  (-0.87, 0.43) | 0.33 |  | .503 |
|  | SES (IMD) | 0.02  (0.00, 0.044) | 0.01 | .13 | .082 |
|  | Sex | 0.01  (-0.08, 0.11) | 0.05 | .02 | .810 |
|  | Age | 0.02  (0.01, 0.03) | 0.01 | .24 | .003 |
|  | Processing Speed | 0.00  (0.00, 0.00) | 0.00 | -.15 | .048 |
|  | Working Memory | 0.05  (0.02, 0.08) | 0.02 | .26 | .001 |
|  | Inhibitory Control | 0.02  (0.01, 0.03) | 0.01 | .21 | .008 |
| **Model 3** | Constant | -0.42  (-1.05, 0.20) | 0.32 |  | .183 |
|  | SES (IMD) | 0.01  (-0.01, 0.03) | 0.01 | .06 | .454 |
|  | Sex | 0.01  (-0.08, 0.10) | 0.05 | .02 | .832 |
|  | Age | 0.02  (0.01, 0.03) | 0.01 | .23 | .002 |
|  | Processing Speed | 0.00  (0.00, 0.00) | 0.00 | -.12 | .109 |
|  | Working Memory | 0.04  (0.01, 0.06) | 0.01 | .19 | .014 |
|  | Inhibitory Control | 0.01  (0.00, 0.02) | 0.01 | .10 | .188 |
|  | Verbal Ability | 0.01  (0.00, 0.01) | 0.00 | .33 | < .001 |

*Note.* Mathematical ability (TEMA) scores were transformed using log10. *R^2^* = .25 for Model 1, Δ *R^2^* = .32 for Model 2, Δ *R^2^* = .40 for Model 3. Pairwise deletion was used for missing data.

For study one, in Model 1, SES and age were significant predictors of mathematical ability, accounting for 25% of variance (*F*_(4,129)_ = 10.56, *p* < .001). Sex and processing speed did not significantly predict mathematical ability. In Model 2, working memory, inhibitory control and processing speed were significant predictors of mathematical ability, overall accounting for 32% of variance in mathematical ability (*F*_(6,127)_ = 11.55, *p* < .001). After introducing these variables in Model 2, SES was no longer a significant predictor, and sex continued to not significantly predict mathematical ability. This suggests that working memory and inhibitory control may be accounting for the socioeconomic gradient in mathematical ability. In Model 3, verbal ability was a significant predictor of mathematical ability. Working memory remained a significant predictor, but inhibitory control and processing speed were no longer significant predictors of mathematical ability. The final model accounted for 40% of variance (*F*_(7,126)_ = 13.39, *p* < .001).

**Table S4.** Results of the Hierarchical Regression Analyses Predicting Mathematical Ability for study two.

|  | **Predictor** | ***b***  ***(95% confidence intervals)*** | ***SE B*** | ***β*** | ***p*** |
| --- | --- | --- | --- | --- | --- |
| **Model 1** | Constant | -5.50  (-20.12, 9.13) | 7.37 |  | .457 |
|  | SES (Mother Education) | 0.85  (0.31, 1.40) | 0.28 | .29 | .003 |
|  | Sex | 1.61  (-0.60, 3.82) | 1.11 | .13 | .152 |
|  | Age | 0.29  (0.16, 0.57) | 0.14 | .19 | .039 |
|  | Processing Speed | 0.00  (-0.01, 0.00) | 0.00 | -.23 | .018 |
| **Model 2** | Constant | -3.54  (-15.49, 8.40) | 6.02 |  | .557 |
|  | SES (Mother Education) | 0.38  (-0.09, 0.84) | 0.24 | .13 | .111 |
|  | Sex | 0.74  (-1.08, 2.57) | 0.92 | .06 | .423 |
|  | Age | 0.13  (-0.11, 0.36) | 0.12 | .83 | .284 |
|  | Processing Speed | 0.00  (-0.01, 0.00) | 0.00 | -.16 | .039 |
|  | Working Memory | 0.92  (0.46, 1.39) | 0.23 | .31 | < .001 |
|  | Inhibitory Control | 0.62  (0.39, 0.86) | 0.12 | .41 | < .001 |
| **Model 3** | Constant | -5.06  (-16.65, 6.53) | 5.84 |  | .388 |
|  | SES (Mother Education) | 0.16  (-0.31, 0.64) | 0.24 | .06 | .499 |
|  | Sex | 0.63  (-1.14, 2.39) | 0.89 | .05 | .482 |
|  | Age | 0.08  (-0.15, 0.30) | 0.11 | .05 | .488 |
|  | Processing Speed | 0.00  (-0.01, 0.00) | 0.00 | -.11 | .171 |
|  | Working Memory | 0.81  (0.35, 1.26) | 0.23 | .27 | < .001 |
|  | Inhibitory Control | 0.54  (0.31, 0.77) | 0.12 | .36 | < .001 |
|  | Verbal Ability | 0.11  (0.03, 0.19) | 0.04 | .25 | .006 |

*Note. R^2^* = .22 for Model 1, Δ *R^2^* = .49 for Model 2, Δ *R^2^* = .53 for Model 3. Pairwise deletion was used for missing data.

For study two, in Model 1, SES, age, and processing speed were significant predictors of mathematical ability, accounting for 22% of variance (*F*(4,96) = 6.63, *p* < .001). Sex was not a significant predictor of mathematical ability. In Model 2, working memory and inhibitory control were significant predictors of mathematical ability, overall accounting for 49% of variance in mathematical ability (*F*(6,94) = 15.00, *p* < .001). After introducing these variables in Model 2, mother’s education and age was no longer significant predictors of mathematical ability, suggesting (as with study one) that working memory and inhibitory control may be accounting for the socioeconomic gradient in mathematical ability. In Model 3, verbal ability was added, which was a significant predictor of mathematical ability. The final model accounted for 53% of variance (*F*(7,93) = 14.90, *p* < .001).

**Testing alternate mediation models**

While the mediation model presented in the manuscript which explores indirect effects with SES as predictor, inhibitory control/ verbal ability as mediators, and mathematical ability as the outcome is theoretically grounded, due to the nature of the concurrent correlational data, it is not possible to disentangle causal directions among variables. We thank an anonymous reviewer for suggesting we supplement these analyses with alternative statistically plausible models which are presented below. Below, we summarize additional analyses that we conducted, in which we test other viable (but non-hypothesized) models as alternative models for comparison in line with the models suggested. To do this, we treated the mediators as outcome variables (Agler & De Boeck, 2017). Details of these models and the accompanying analyses are presented here for full transparency. The additional analyses show that our hypothesized model does explain more variance than the alternate models.

**Study 1:**

The first alternative mediation tested whether inhibitory control and mathematical ability mediated the relation between SES and verbal ability. The model was fit with SES as the predictor; inhibitory control and mathematical ability as indirect effects; and verbal ability as the outcome variable. Processing speed, age, and sex were included as covariates (Figure S3). In the total effect model, SES had a significant positive effect on verbal ability (β = .35, p < .001). In the mediated model, SES had a significant positive effect on inhibitory control (β = .27, p = .002) and mathematical ability (β = .23, p = .006). Inhibitory control (β = .31, p = .001) and mathematical ability (β = .30,p = .001) had significant positive effects on verbal ability. The results of the bootstrapping procedure revealed that the indirect effect through inhibitory control (95% CI [0.03, 0.16]) and the indirect effect through mathematical ability (95% CI [0.02, 0.16]) were significant, as the CIs did not pass through zero. The CIs indicated that inhibitory control and mathematical ability mediate the relation between SES and verbal ability. Pairwise contrasts of the indirect effects through inhibitory control and mathematical ability (95% CI [-0.11, 0.08]) indicated that the paths did not differ significantly from each other, as the CIs passed through zero.


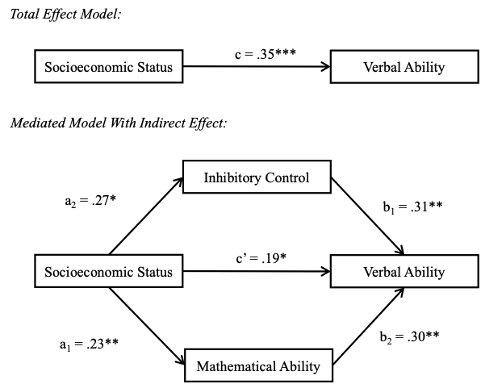


Figure S3. Mediation model showing that the relation between SES (IMD) and verbal ability is indirectly explained by inhibitory control and mathematical ability, controlling for processing speed, age and sex. Standardized beta weights are given. *p <.05, **p < .01, ***p < .001.

The second alternative mediation tested whether verbal ability and mathematical ability mediated the relation between SES and inhibitory control. The model was fit with SES as the predictor; verbal ability and mathematical ability as indirect effects; and inhibitory control as the outcome variable. Processing speed, age, and sex were included as covariates (Figure S4). In the total effect model, SES had a significant positive effect on inhibitory control (β = .27, p = .002). In the mediated model, SES had a significant positive effect on verbal ability (β = .35, p < .001) and mathematical ability (β = .23, p = .006). Verbal ability (β = .33, p < .001) and mathematical ability (β = .24, p = .009) had significant positive effects on inhibitory control. The results of the bootstrapping procedure revealed that the indirect effect through verbal ability (95% CI [0.05, 0.20]) and the indirect effect through mathematical ability (95% CI [0.01, 0.13]) were significant, as the CIs did not pass through zero. The CIs indicated that verbal ability and mathematical ability mediate the relation between SES and inhibitory control. Pairwise contrasts of the indirect effects through inhibitory control and mathematical ability (95% CI [-0.16, 0.03]) indicated that the paths did not differ significantly from each other, as the CIs passed through zero.


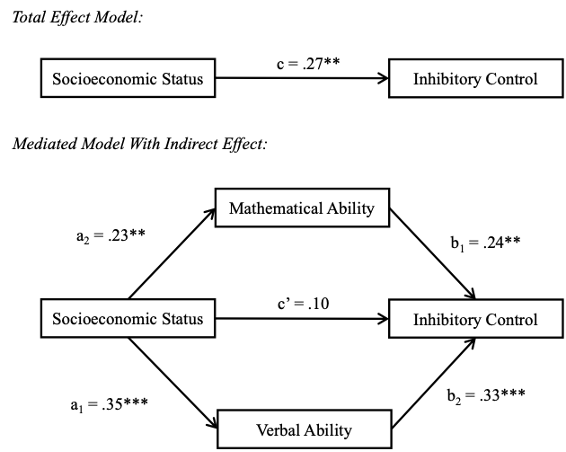


Figure S4. Mediation model showing that the relation between SES (IMD) and inhibitory control is indirectly explained by verbal ability and mathematical ability, controlling for processing speed, age and sex. Standardized beta weights are given. *p <.05, **p < .01, ***p < .001.

**Study 2:**

The first alternative mediation tested whether inhibitory control and mathematical ability mediated the relation between SES and verbal ability. The model was fit with SES as the predictor; inhibitory control and mathematical ability as indirect effects; and verbal ability as the outcome variable. Processing speed, age, and sex were included as covariates (Figure S5). In the total effect model, SES had a significant positive effect on verbal ability (β = .38, p < .001). In the mediated model, SES had a significant positive effect on inhibitory control (β = .27, p = .009) and mathematical ability (β = .29, p = .003). Mathematical ability (β = .34, p = .001) had a significant positive effect on verbal ability, but inhibitory control did not have a significant effect on verbal ability (β = .09, p = .363). The results of the bootstrapping procedure revealed that the indirect effect through inhibitory control (95% CI [-0.02, 0.10]) was significant. The indirect effect through mathematical ability (95% CI [0.03, 0.20]) was significant, as the CIs did not pass through zero. The CIs indicated that mathematical ability mediates the relation between SES and verbal ability, but that inhibitory control does not. Pairwise contrasts of the indirect effects through inhibitory control and mathematical ability (95% CI [-0.02, 0.21]) indicated that the paths did not differ significantly from each other, as the CIs passed through zero.

Figure S5. Mediation model showing that the relation between SES (IMD) and verbal ability is indirectly explained by inhibitory control and mathematical ability, controlling for processing speed, age and sex. Standardized beta weights are given. *P <.05, **p < .01, ***p < .001.

The second mediation model tested whether verbal ability and mathematical ability mediated the relation between SES and inhibitory control. A mediation analysis with two indirect effects was conducted. The model was fit with SES as the predictor; verbal ability and mathematical ability as indirect effects; and inhibitory control as the outcome variable. Processing speed, age, and sex were included as covariates (Figure S6). In the total effect model, SES had a significant positive effect on inhibitory control (β = .27, p = .009). In the mediated model, SES had a significant positive effect on verbal ability (β = .38, p < .001) and mathematical ability (β = .29, p = .003). Mathematical ability (β = .48, p < .001) had a significant positive effect on inhibitory control, but verbal ability (β = .10, p = .363) did not. The results of the bootstrapping procedure revealed that the indirect effect through verbal ability (95% CI [-0.04, 0.13]) was not significant. The indirect effect through mathematical ability (95% CI [0.05, 0.27]) was significant, as the CIs did not pass through zero. The CIs indicated that mathematical ability mediated the relation between SES and inhibitory control. Pairwise contrasts of the indirect effects through inhibitory control and mathematical ability (95% CI [-0.26, 0.02]) indicated that the paths did not differ significantly from each other, as the CIs passed through zero.

Figure S6. Mediation model showing that the relation between SES (IMD) and verbal ability is indirectly explained by inhibitory control and mathematical ability, controlling for processing speed, age and sex. Standardized beta weights are given. *P <.05, **p < .01, ***p < .001.
